# Case Report: Intranasal esketamine combined with a form of generative artificial intelligence in the management of treatment-resistant depression

**DOI:** 10.3389/fpsyt.2025.1536232

**Published:** 2025-08-14

**Authors:** Alexandre Fraichot, Sophie Favre, Hélène Richard-Lepouriel

**Affiliations:** ^1^ Mood and Anxiety Disorder Unit, Psychiatric Specialties Service, Department of Psychiatry, Geneva University Hospital, Geneva, Switzerland; ^2^ Department of Psychiatry, University of Geneva, Geneva, Switzerland

**Keywords:** intranasal esketamine, artificial intelligence, treatment-resistant depression, case report, depression

## Abstract

**Introduction:**

Intranasal Esketamine is an effective rapid-acting antidepressant currently used to treat treatment-resistant depression. Artificial intelligence is another emerging tool in medicine, but little is known about the effectiveness of combining these innovations in psychiatry.

**Methods:**

This case report presents the outcome of a 37-year-old patient who received intranasal Esketamine treatment (84 mg) and utilized artificial intelligence (ChatGPT-4) to generate images and interpretations of his experiences with dissociation. This process was conducted in the presence of a nurse who assessed and supported the patient. The Montgomery–Åsberg Depression Rating Scale (MADRS) was used to measure the severity of depression at the beginning of each session.

**Results:**

The patient achieved remission from depression, with MADRS scores declining by 50% in the third session, and the scores indicated mild depression or euthymia in the eight subsequent sessions. The patient reported that incorporating artificial intelligence-generated images and interpretations helped him create a timeline of his experiences at the end of each session.

**Discussion:**

This case report highlights the potential effectiveness of combining intranasal Esketamine treatment with generative artificial intelligence images and interpretations as part of an integration process. It also emphasizes the importance of having a nurse present to support the process. Further research is needed to determine which patients may benefit most from this combined treatment approach.

## 1 Introduction

Intranasal Esketamine has gained attention following its approval by the US Food and Drug Administration (2019) as well as by the European Medicines Agency (2021) to treat treatment-resistant depression, in combination with a selective serotonin reuptake inhibitor or serotonin and norepinephrine reuptake inhibitor. The REAL-ESK study suggests that combining Esketamine with Vortioxetine may benefit patients unresponsive to standard treatments (1). A systematic review of ten studies supports Esketamine’s ability to reduce depressive symptoms (2), with tolerable side effects such as nausea, dizziness, dissociation, and headache, mostly occurring around treatment sessions (3). However, long-term effects after discontinuation remain inconsistent, necessitating more robust randomized controlled trials (4). Some patients show delayed clinical responses at six months, with further symptom improvement even after stopping treatment for clinical reasons (5).

Efficacy tends to increase at doses above 28 mg, with optimal results seen between 56 and 84 mg (6). Continuation beyond the initial 4-week induction phase is recommended to maintain stability and prevent relapse (7). Esketamine, a derivative of ketamine, is a dissociative anesthetic that induces altered states of consciousness by disrupting top-down processing in the brain’s higher association cortex (8). These experiences—altered self-perception, emotional expansion, and increased connectedness—have been described by patients as therapeutically valuable (9). Integration of these experiences is crucial; without proper reflection, such effects may not yield sustained benefits (10).

Esketamine-induced dissociation may be particularly effective in cases of depersonalized depression, helping to restore emotional responsiveness by disrupting rigid patterns of thought and behavior (11). Misconceptions around Esketamine, including its dissociative effects and potential for addiction, persist. However, evidence indicates these effects are transient and generally not central to its antidepressant action (12).

Artificial intelligence (AI) is increasingly integrated into mental health care to enhance diagnostic accuracy, optimize clinical workflows, and facilitate the visualization of complex clinical data (13–17). In psychiatry, AI is applied for screening, symptom monitoring, and the development of personalized treatment plans, including conditions such as depression and treatment-resistant depression. AI-generated imagery has emerged as a novel adjunct in psychiatric care; these tools produce personalized visuals based on user input, enabling patients to explore and process emotions, memories, and fears—thereby supporting therapy through visualization and self-reflection (18).

Beyond its therapeutic uses, AI can analyze clinical data to predict treatment responses, including to interventions like intranasal Esketamine, thus offering opportunities for individualized care (19). Machine-learning models have demonstrated potential in forecasting treatment outcomes, which may support more targeted and efficient use of Esketamine in clinical settings (19, 20).

However, the integration of AI into mental health care presents notable challenges, including algorithmic bias, risks to data privacy, and ethical considerations related to incorporation into medical records (18, 20). For instance, while generative platforms such as ChatGPT-4 can produce emotionally resonant imagery, they may also perpetuate stereotypes, underscoring the necessity for robust ethical oversight (21, 22).

Although AI cannot replace human-led therapy, it can meaningfully enhance therapeutic processes when embedded within professionally guided frameworks. When implemented responsibly, AI—alongside interventions like Esketamine—may represent a promising frontier in the treatment of treatment-resistant depression.

AI-generated imagery is widely accessible and can be integrated into mental health interventions, helping individuals express emotions and engage in self-reflection. Tools like ChatGPT-4 (21) can create personalized visuals based on user input. While GPT-4 improves comprehension and warmth over GPT-3.5, it also shows an increased tendency toward stereotyping, highlighting the need for careful use in therapeutic settings (22).

This case study presents the evolution of a patient over 11 sessions of intranasal Esketamine administration combined with an antidepressant medication and enhanced by AI-generated images and interpretations. The patient contributed to the protocol by introducing ChatGPT-4 to journal his experiences directly after the Esketamine administration. This case report illustrates the emerging use of AI in contemporary clinical practice.

## 2 Case description

This case report used the CARE Checklist guidelines (23).

Edgar (alias), a 37-year-old man with a history of depression and anxiety, has experienced multiple depressive episodes, with his most severe occurring in 2018 when he was hospitalized, and in 2023. There is a family history of depressive disorders among his maternal relatives. There were no suicide attempts in his family, and he has never attempted suicide himself, although he has experienced suicidal ideation during episodes of depression. He has undergone various treatments, including Trazodone (prolonged formulation, 150 mg) and Bupropion 300 mg; and Pregabalin (25 mg, taken twice daily). He also received treatment with intranasal Esketamine (14 mg) combined with Escitalopram (10 mg) at a university hospital participating in a research program focused on the efficacy of low-dose Esketamine, where he had participated to five sessions and tolerated the low dosage well. However, a potentiation strategy using non-antidepressant medication was lacking to confirm any criteria for drug resistance. To address this, Quetiapine XR (50 mg) was introduced to evaluate whether this potentiation strategy would demonstrate total or partial efficacy. Since this approach did not yield the desired results, treatment with intranasal Esketamine was reconsidered.

### 2.1 Clinical measures:

Various scales assess depression severity. The Beck Depression Inventory (BDI) is a self-reported scale and may be affected by patients’ inability to assess their symptoms accurately (24). The Hamilton Depression Rating Scale (HDRS) includes non-mood symptoms like anxiety which can persist independently of mood improvement, potentially leading to an overestimation of depression severity after treatment. Additionally, the HDRS may not effectively capture the early response to treatment (25, 26). In Esketamine treatment protocols, the Montgomery–Åsberg Depression Rating Scale (MADRS) (27, 28) is preferred due to its sensitivity to early improvements in depression severity. This clinician-administered scale consists of 10 items rated from 0 to 6, with total scores classifying depression from mild (7–19) to severe (≥35). A two-point improvement is considered clinically relevant (29).

Additionally, blood pressure is monitored, with normal values for males aged 18–39 being 119/70 mm Hg (30).

### 2.2 Procedure

Esketamine nasal spray, available under a restricted distribution program, is used alongside another antidepressant in two phases: a 4-week induction phase (twice weekly) and a maintenance phase (weekly for responders). Patients are monitored for at least two hours post-administration for side effects, including nausea, dissociation, sedation, and cognitive impairment. Severe risks include abuse or misuse, suicidal thoughts, and elevated blood pressure.

Before treatment, patients undergo a medical check-up, including blood pressure monitoring and an ECG, and are advised against driving until after a whole night’s rest. Esketamine is contraindicated in patients with hypersensitivity to Esketamine/Ketamine, vascular aneurysms, arteriovenous malformations, or intracerebral hemorrhage (31).

In this case study, a senior psychiatrist (HRL) diagnosed the patient with recurrent depression (ICD-11) (32) with comorbid anxiety. The MADRS score was 23/60, indicating moderate depression, with no psychiatric or somatic comorbidities. The psychiatrist carefully monitored side effects after each Esketamine session.

Esketamine was first administered at 28mg and, as it was well tolerated, was then rapidly prescribed at 84 mg and remained with good tolerability and clinical response. The patient was also prescribed Quetiapine XR (50 mg) (**Table 1**).

**Table 1:** Scheme of the treatment.

Session	1	2	3	4	5	6	7	8	9	10	11
IN Esketamine (mg)	28	56	84	84	84	84	84	84	84	84	84

2 sessions per week during the first month.

During weekly sessions, the nurse (AF) administered intranasal Esketamine, ensured patient comfort, and monitored effects. He assessed severity of depression using the MADRS, provided feedback, and discussed any lingering feelings or dissociative experiences from previous sessions, and was prepared to manage side effects, particularly anxiety. The patient introduced the use ChatGPT-4 to generate images representing his experiences from the first session on, dictating his visions until the AI-created images aligned with them. He also reflected on these images using ChatGPT’s interpretations after the sessions.

At the time, Edgar was not receiving psychotherapy, and no psychologists were involved in the process.

Ethical review was not required for this single case study, as it did not constitute research involving human subjects under prevailing ethical guidelines. The report described routine clinical care without systematic investigation or experimental intervention, and the case did not involve sensitive information. The patient’s anonymity was preserved, and informed consent was obtained for the publication of both clinical data and the accompanying figure.

### 2.3 Treatment outcomes

The evolution of the MADRS scores is presented in **Table 2**.

**Table 2:** MADRS scores.

Session	1	2	3	4	5	6	7	8	9	10	11
MADRS scores	23	13	9	10	6	8	8	8	4	13	9
Severity of depression	moderate	mild	mild	mild	euthymia	mild	mild	mild	euthymia	mild	mild

2 sessions per week during the first month. A total score ranging from 0 to 6 indicates that the patient is in the normal range (no depression), a score ranging from 7 to 19 indicates “mild depression,” 20 to 34 indicates “moderate depression,” a score of 35 and greater indicates “severe depression.”

During the third Esketamine session, Edgar’s MADRS score dropped by over 50% (from 23 to 9). A reduction of 50% or more in depressive symptoms is typically regarded as a response to antidepressants in clinical trials (33). The severity of depression fluctuated between mild depression and euthymia throughout the treatment. Blood pressure remained stable (mean: 127.2/83.5 mm Hg), and side effects such as mild nausea and transient increases in blood pressure were mild and temporary (grade ≥ 2) (34), typically peaking within 40 minutes post-administration.

Dissociative effects emerged approximately 10 minutes after inhalation and lasted about an hour. Edgar used this time to document his experiences via ChatGPT-4, generating images that reflected his perceptions. He also discussed this with the nurse, integrating the other altered perceptions he had experienced, such as feelings of warmth, tactile sensations, and a sense of detachment.

**Figure 1:**
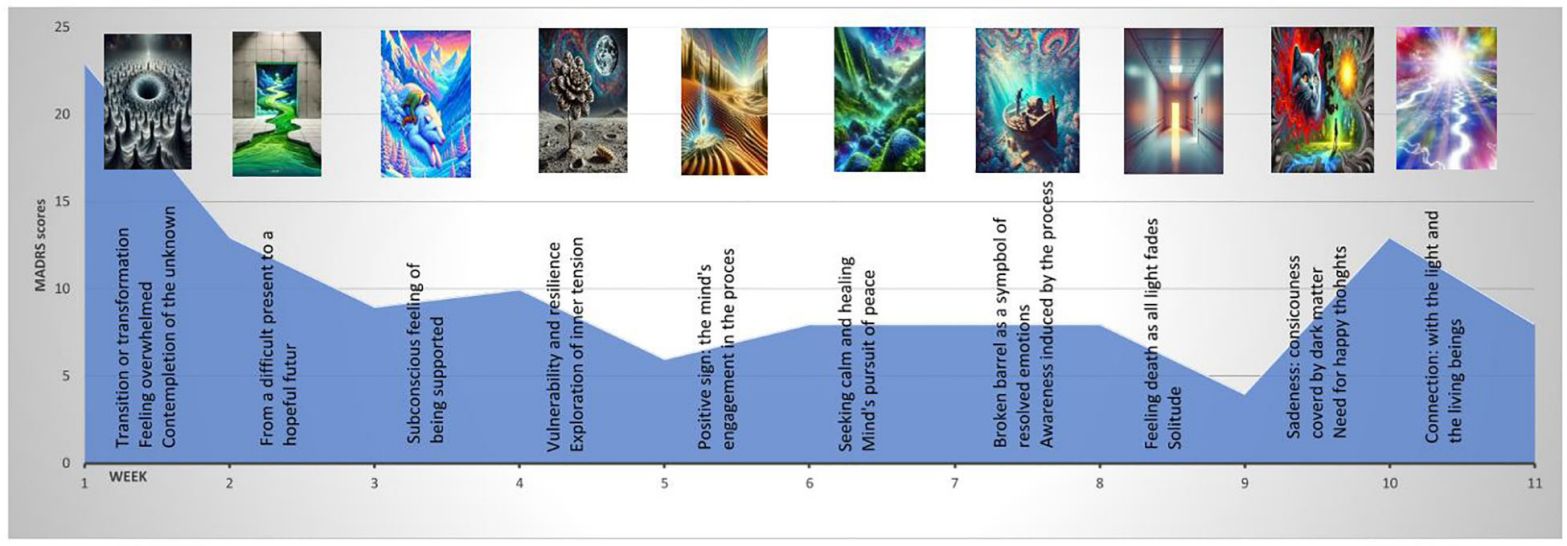
Timeline of the process.

In session one, he experienced anxiety and disturbing thoughts. In **Figure 1**, the first image created by ChatGPT-4 during the first session is darker than the following ones. Then, the AI-generated images, which were colorful and dreamlike, depicted themes such as nature, water, space, and abstract shapes. Edgar found most images pleasant, except for the first (darker) and eighth (hospital-like, evoking a sense of dying). The third session was significant, as he envisioned an entity from which he could seek support.

For **Figure 1**, the patient selected images and interpretations generated with ChatGPT-4. The timeline helped him describe his journey as “a struggle with inner tensions evolving into peace of mind and a feeling of connection.” **Figure 1** presents the timeline with the MADRS scores, the images, and the AI-generated interpretation during the first 11 sessions of the treatment.

Edgar valued the AI-generated timeline of his sessions, seeing it as a visual representation of progress that helped him navigate anxiety and depression. He observed that this timeline allowed him to revisit his prior experiences whenever he feels anxious or depressed. He said, “I need to see the progress and representative visual data. I can look back and see where I’m headed and compare my current state with various other moments.

While acknowledging that ChatGPT-4 is algorithm-based, he felt the images reflected truthfully and carefully his unique perspective. That differed from interactions with humans and caregivers, where components that were not his could be added.

## 3 Patient perspective

The patient expressed that Esketamine allowed him to access a mental space where he could explore important questions about his history and suffering, which had previously been inaccessible to him. Integrating AI into his treatment felt natural, as AI is a regular part of his daily life. Specifically, using ChatGPT-4 helped him create a timeline of his experiences, which he found valuable when managing feelings of anxiety or depression. He noted that this timeline reflected a shift from darker experiences to more colorful ones, representing his assessment of his evolution during treatment. The patient also appreciated the consistency of having the same nurse throughout his treatment, emphasizing how important it was to have someone present during moments of vulnerability.

## 4 Discussion

In this case report, treatment-resistant depression was treated with intranasal Esketamine, which is considered a “safe, effective, and rapid-acting option” for persons with treatment-resistant depression (33). The MADRS scores indicated a rapid remission that may be associated with factors related to the medication, the patient’s clinical history, and the patient’s age. Research has shown that antidepressant responses tend to be less robust in younger (≥ 21 years old) and older (≤ 35 years old) patients (35), and this patient falls within the age range that generally benefits the most from these treatments. Additionally, in older patients, specific symptoms (such as sadness, appetite changes, and concentration difficulties) can affect residual symptoms in those severely resistant to treatment (36), which was not the case here.

This case report explores the combination of intranasal Esketamine and AI, specifically ChatGPT-4, in the treatment of a patient with treatment-resistant depression. The patient used AI to document and interpret his experiences after inhaling Esketamine, which helped him to express his thoughts and emotions. This form of journaling, supported by the nurse’s empathy, created an integrative therapeutic approach that strengthened the patient-nurse relationship and enhanced the therapeutic process. AI productions also facilitated supportive discussions through storytelling, which, in this case, was broadly an act of sharing experiences and ideas with the nurse, which contributed to helping the patient organize his thoughts and process emotions and personal experiences (37). The patient also shared his AI-generated images with his partner and friends, which may have further enhanced his emotional processing and experiences of belonging.

This patient’s adjunction of ChatGPT’s images and interpretation to monitor his experiences during intranasal Esketamine treatment aligns with current practices that currently investigate real-time monitoring and predictive analytics, precision medicine, and personalized treatment, as well as the role of ChatGPT in telemedicine (38). However, AI is a double-edged sword when growing from personal to medical fields and integrating AI technologies into medical research poses significant ethical challenges. AI systems may compromise patient data privacy and confidentiality, perpetuate biases if trained on non-diverse or nonrepresentative datasets, obscure accountability due to the opacity of AI models, and produce unreliable outputs that could mislead researchers and clinicians (39, 40). ChatGPT-4 is also used as a tool in the medical publication landscape. Experts emphasized human corrections to the analysis produced by this AI tool (41).

This study presents some limitations. In this study, only the primary outcome (depression) was regularly assessed. The lack of measures of anxiety to objectively assess how anxiety evolved during the treatment, as well as the lack of measures of dissociation (42) within sessions that would have informed the severity of dissociation. These data, along with the data on the severity of depression and the measure of long-term follow-up, would have brought a more thorough picture of the process. Finally, one limitation of case report study designs is the impossibility of attributing the treatment’s efficacy to one of the interventions or the combination of both. Symptomatic improvement in depression may result from antidepressants (biological effects), nursing support (emotional stability), or journaling (cognitive processing). However, their effects likely interact. A randomized controlled trial (2x2x2 factorial) is the best procedure to determine whether antidepressant treatment, nursing support, or journaling independently contribute to symptomatic improvement in depression. If similar improvements occur across intervention groups, psychosocial factors or the placebo effect may be more influential than medication. The best outcomes may come from combining biological, psychological, and social interventions rather than relying on a single factor. Moreover, single-case studies have limited generalizability, lack control groups, and may be influenced by bias or individual variability. They make it difficult to establish causation, requiring cautious interpretation and validation through larger studies. Given these limitations, conclusions should be drawn cautiously, avoiding overinterpretation based on limited data. Further research is needed to ascertain the enhanced effectiveness of combining intranasal Esketamine and ChatGPT on the outcome.

This case report indicates that intranasal Esketamine can be combined with AI to document ongoing experiences of dissociation and enhance the therapeutic process. This case report provides insights into the potential effectiveness of merging these two innovative approaches in treating individuals with treatment-resistant depression. Both intranasal Esketamine and AI are readily available resources, and their combination could prove cost-effective in the long run. The presence of a nurse can consist of supporting this journaling process, which might otherwise be limited or misdirected. The individual contributions of each component, as well as their interactions, necessitate further investigation. These multimodal treatments are vital in complex or severe mental health conditions, where tailored and collaborative approaches can improve outcomes. Additional research is needed to establish the broader applicability of the findings from this specific case.

## Data Availability

The original contributions presented in the study are included in the article/supplementary material. Further inquiries can be directed to the corresponding author/s.
